# Targeting beta-Catenin signaling to induce apoptosis in human breast cancer cells by z-Guggulsterone and Gugulipid extract of *Ayurvedic* medicine plant *Commiphora mukul*

**DOI:** 10.1186/1472-6882-13-203

**Published:** 2013-08-03

**Authors:** Guoqin Jiang, Xiao Xiao, Yan Zeng, Kalyanam Nagabhushanam, Muhammed Majeed, Dong Xiao

**Affiliations:** 1 Department of General Surgery, The 2nd Affiliated Hospital of Soochow University, 1055 SanXian Road, Suzhou 215004, Jiangsu, China; 2 Department of Urology, and University of Pittsburgh Medical College, University of Pittsburgh, Shadyside Medical Center, Suit G30, 5200 Centre Avenue, Pittsburgh PA 15232, USA; 3 University of Pittsburgh Cancer Institute, University of Pittsburgh Medical College, University of Pittsburgh, Shadyside Medical Center, Suit G30, 5200 Centre Avenue, Pittsburgh, PA 15232, USA; 4 Sabinsa Corporation, 20 Lake Drive, East Windsor, NJ 08520, USA

**Keywords:** Gugulipid, Guggulsterones, Apoptosis, Beta-Catenin, Breast cancer

## Abstract

**Background:**

z-Guggulsterone (z-Gug) and Gugulipid (GL) have been used to treat a variety of ailments. We now report their anti-cancer effect and mechanism against human breast cancer.

**Methods:**

Using the human estrogen receptor-positive (MCF-7) and triple-negative (MDA-MB-231) breast cancer cells as well as the normal human mammary epithelial cell line (HMEC), we evaluated the anti-breast-cancer efficacy and apoptosis inducing activity of GL. We determined the cellular and molecular mechanism of GL-inhibited breast cancer cell growth.

**Results:**

GL significantly inhibited growth of MCF-7 and MDA-MB-231 cells with an IC_50_~2 μM at pharmacologically relevant concentrations standardized to its major active constituent z-Gug. The GL-induced growth inhibition correlated with apoptosis induction as evidenced by an increase in cytoplasmic histone-associated DNA fragmentation and caspase 3 activity. The GL-induced apoptosis was associated with down-regulation of the β-Catenin signaling pathway. The decreased expression of Wnt/β-Catenin targeting genes, such as cyclin D1, C-myc and survivin, and the inhibition of the activity of the transcription factor (T-cell factor 4, TCF-4) were observed in GL-treated breast cancer cells. The GL treatment resulted in a significant reduction of β-Catenin /TCF-4 complex in both of the cancer cells. The GL-induced apoptotic cell death was significantly enhanced by RNA Interference of β-Catenin and TCF-4. On the other hand, the normal human mammary epithelial cell HMEC, compared with the human breast cancer cells, is significantly more resistant to growth inhibition and apoptosis induction by GL.

**Conclusion:**

The present study indicates that the β-Catenin signaling pathway is the target for GL-induced growth inhibition and apoptosis in human breast cancer.

## Background

Despite significant advances toward targeted therapy and screening techniques, breast cancer continues to be the leading cause of cancer-related deaths and the most frequently diagnosed cancer among women in the USA and worldwide [1]. The clinical utility of ER antagonists is often limited by side effects [1]. Thus, the identification of novel agents that can suppress growth of both ER-positive and ER-negative human breast cancers and are still relatively safe is highly desirable.

Gugulipid (GL, guggul, guggal, or gugul lipid) is the ethyl acetate extract of the gum guggul resin (raw material) harvested directly from the *Commiphora mukul* tree (family name: Burseraceae; synonyms: Hook, Bandari, *Balsamodendron mukul*, and *Commiphora Wightii*). GL is a highly valued botanical medicine that has been safely used for thousands of years in the Indian Ayurvedic medicine for the treatment of different ailments, including lipid disorders, rheumatoid arthritis, ulcers, osteoarthritis, bone fractures, epilepsy and obesity [2-10]. In 1986, GL was granted approval in India for marketing as a lipid-lowering drug (Indian Pharmacopeia 2007: pgs. 2038–2040). Several products of standardized formulations of *Commiphora mukul* are already in human use as cholesterol-lowering agents [5-8]. The z- and E-forms of guggulsterone (Gug, 4,17 [11]-pregnadie-3, 16-dione) have been identified as major active components of GL [2-10]. Numerous studies suggest that many edible phytochemicals have cancer chemopreventive and chemotherapeutic potential [12]. The evidences of the anti-cancer activity of Gugs were provided by us and other laboratories [11,13-24]. We were the first to investigate the inhibitory effect of Gug on the growth of the human prostate cancer cells [13-16]. The results have shown that z-Gug significantly inhibits the proliferation of PC-3, LNCaP and DU145 human prostate cancer cells, but not that of the normal human prostate epithelial cell line PrEC [14-16]. Based on these data, we hypothesized that GL might be more effective in the growth inhibition of prostate cancer cells because it contains a number of steroids, including the two isomers z- and E-Gugs. Therefore, we investigated the anti-cancer potential of GL in human prostate cancer cells [13]. Our data were the first to show that GL has a stronger anti-cancer potential in human prostate cancer cells than z-Gug, one of its active constituents, as evidenced by greater inhibition of cell growth [13]. It is reported that treatment with GL (3 μmol standardized to z-Gug, daily for 3 weeks) resulted in the enhancement of cetuximab activity in the xenograft model of head and neck cancer [20]. The Gugs-mediated suppression of cancer cell proliferation has also been reported in head and neck cancer cells [20], leukemia cells [11,22], lung cancer cells [22], human breast cancer cells [19], skin cancer cells [21], and colon cancer cells [23]. Gug treatment inhibited angiogenesis *in vitro* and *in vivo* to block prostate and colon cancer growth [14,23]. In our present studies, we were the first to report the anti-cancer effect and mechanism of GL on human breast cancer cells.

## Methods

### Reagents

Derived from the gum guggul resin (gum guggul) in the soft bark ducts of the *Commiphora mukul* tree, GL is a registered product of Sabinsa Corporation (East Windsor, NJ, USA, Registration date: July 21, 1992; US Patent# 6436991 B1). We previously described a manufacturing flow chart for the production of GL from gum guggul resin [2]. The standardization of GL was performed by high-performance liquid chromatography (HPLC, 2). GL contains ~3.75% z-Gug and is standardized to z-Gug (μM) [2]. The GL was stored at 4°C and found to be stable for at least 6months. The *z*-Gug was from Steraloids (Newport, RI, USA). The reagents for cell culture including medium, penicillin and streptomycin antibiotic mixture, and fetal bovine serum were purchased from Invitrogen (Carlsbad, CA, USA). The ELISA kit for the quantitation of cytoplasmic histone-associated DNA fragmentation was from Roche Diagnostics (Mannheim, Germany). The intracellular staining/conjugated cleaved caspase-3 (Asp175) antibody (Alexa Fluor®488 conjugate) for the determination of caspase 3 activity by Flow cytometry was from cell Signaling (Danvers, MA, USA). The SurveyorTM IC Human total β-Catenin Immunoassay kit was from R&D Systems (Minneapolis, MN, USA). The anti-β-Catenin antibody was from Invitrogen, the antibodies against Cyclin D1, C-Myc, poly-(ADP-ribose)-polymerase (PARP) and TCF-4 were from Santa Cruz Biotechnology (Dallas, TX, USA), the antibody against Survivin was from Novus Biologicals, the antibody against α-Tubulin was from Sigma, and the anti-actin antibody was from Oncogene Research Products (San Diego, CA, USA). The general caspase inhibitor Z-VAD(oMe)-FMK (Z-VAD) was from Enzyme Systems (Dublin, CA, USA).

### Cell culture and cell survival assays

The MDA-MB-231 and MCF-7 cell lines were purchased from the American Type Culture Collection (Manassas, VA, USA) and maintained by following the supplier’s recommendations. The normal human mammary epithelial cell line (HMEC) was procured from Lonza (Walkersville, MD, USA) and cultured in the epithelial cell basal medium (Lonza). Each cell line was maintained at 37C in an atmosphere of 95% air and 5% CO_2_. Stock solution of GL and z-Gug were prepared in DMSO and an equal volume of DMSO (final concentration 0.1%) was added to the controls. The effect of GL and z-Gug on cell viability was determined by the colonogenic survival assay and trypan blue dye exclusion assays as described by us previously [13,25]. For the colonogenic survival assay, cells (1.5×10^5^) were plated in 6-well-plates for incubation overnight and were then treated with 0.1% DMSO (control group) or 1, 2.5 and 5 μM GL for 24 h. The treated cells were re-seeded in 6-well plates (500 cells/well) in complete medium without the drug. The medium were changed every two days. After culturing for 12days, the cells were fixed and stained with 0.5% crystal violet in 20% MeOH for colony counting.

### Detection of apoptosis

Apoptosis induction by GL was assessed by the analysis of cytoplasmic histone-associated DNA fragmentation, which has emerged as a highly sensitive and reliable technique for the quantitation of apoptotic cell death. The cytoplasmic histone-associated DNA fragmentation was determined as described by us previously [13,26-28]. In some experiments, cells were pretreated with 40 μM pan-caspase inhibitor Z-VAD for 2hours before GL treatment and assessment of apoptotic cell death described as our previous study [16].

### Determination of caspase-3 activation [13,28]

The activation of caspase-3 was determined by flow cytometry using a kit from Cell Signaling Technology. Briefly, the cells (3×10^5^) were plated in T25 flasks and allowed to attach by overnight incubation. The cells were then treated with Me_2_SO (control) or the desired concentrations of GL for the specified time periods. Subsequently, the cells were collected by trypsinization and processed for the flow cytometric analysis of caspase-3 activation according to the manufacturer’s instructions.

### Measurement of β-Catenin level

Experiments to determine the effects of GL on β-catenin level were carried out using the Surveyor™ IC Human total β-Catenin Immunoassay kit (R&D Systems) following the manufacturer’s instructions. In brief, the cells (2×10^6^) were seeded in 75 cm^2^ flasks and allowed to attach by overnight incubation. The cells were treated with DMSO (control) or 2.5 and 5 μM GL or 20 and 40 μM z-Gug for 24 h. The cells were collected, washed with phosphate-buffered saline, and lysed by the specific lysis buffer provided by the kit. The lysate were used to determine the β-Catenin level.

### Preparation of cell extracts

Whole cell extracts, nuclear extracts (Nuc), and cytosolic extracts (Cyto) were prepared essentially as described by us previously [28]. NE-PER® nuclear and cytoplasmic extraction reagents (Pierce product) were used to prepare cytosolic and nuclear extracts. Briefly, the treated cells were placed on ice, the medium was removed, and the cells were washed once with cold PBS. The cells were then scraped off the dish and collected by centrifugation. Cell pellets were used to prepare the cytosolic and nuclear extracts following the product instructions. The protein concentration was determined using the Bio-Rad Protein Assay reagent with bovine serum albumin as the standard.

### Immunoblotting

The lysate proteins were resolved by 6–12.5% sodium dodecyl sulfate polyacrylamide gel electrophoresis and transferred onto the membrane. Immunoblotting was performed as described by us previously [26-28]. The blots were stripped and re-probed with the anti-actin antibody to correct for differences in protein loading. Change in the protein level was determined by densitometric scanning of the immunoreactive band and corrected for actin loading control. Immunoblotting for each protein was performed at least twice using independently prepared lysate proteins.

### RNA interference of β-catenin and TCF-4

The cells (1×10^5^) were seeded in six-well plates and allowed to attach by overnight incubation. The cells were transfected with 200 nM nonspecific control-siRNA (QIAGEN, Cambridge, MA, USA, sequences: sense: 5’-UUCUCCGAACGUGUCACGU-3’, antisense: 5’-ACGUGACACGUUCGGAGAA-3’) or β-Catenin (sc-44252, Santa Cruz, Dallas, TX, USA, sequences: sense: 5’-CUCAGUCCUUCACUCAAGA-3’, antisense: 5’-UCUUGAGUGAAGGACUGAG-3’) or TCF-4 (sc-43525, Santa Cruz, Dallas, TX, USA, sequences: sense: 5’-CUGAGUCCUUCACUCAAGA-3’, antisense: 5’-UCUUGAGUGAAGGACUGAG-3’ ) -targeted siRNA using Oligofectamine (Invitrogen, Grand Island, NY, USA) according to the manufacturer’s recommendations. Twenty-four hours after transfection, the cells were treated with DMSO (control) or 5 μM GL for specified time period. The cells were collected, washed with phosphate-buffered saline, and processed for immunoblotting or analysis of cytoplasmic histone-associated DNA fragmentation as described previously [26-28].

### Immunoprecipitation analyses of the interaction between β-Catenin and TCF-4 in human breast cancer cells

Aliquots containing 200 μg of total lysate protein from MCF-7 and MDA-MB-231 cells that were treated with DMSO (control) or GL were incubated overnight at 4C with 5 μg of anti-β-Catenin antibody. Protein G–agarose beads (50 μL; Santa Cruz Biotechnology, USA) were then added to each sample, and the incubation was continued for an additional 2 h at 4C. The immunoprecipitates were washed five times with lysis buffer and subjected to SDS-PAGE followed by immunoblotting using anti-TCF-4 antibody.

### Statistical analysis

Statistical significance of difference in measured variables between control and treated groups was determined by t*-*test or one-way ANOVA. Difference was considered significant at *P*<0.05.

## Results

### GL inhibited human breast cancer cell growth but not normal human mammary epithelial cell line HMEC

**Figure 1:**
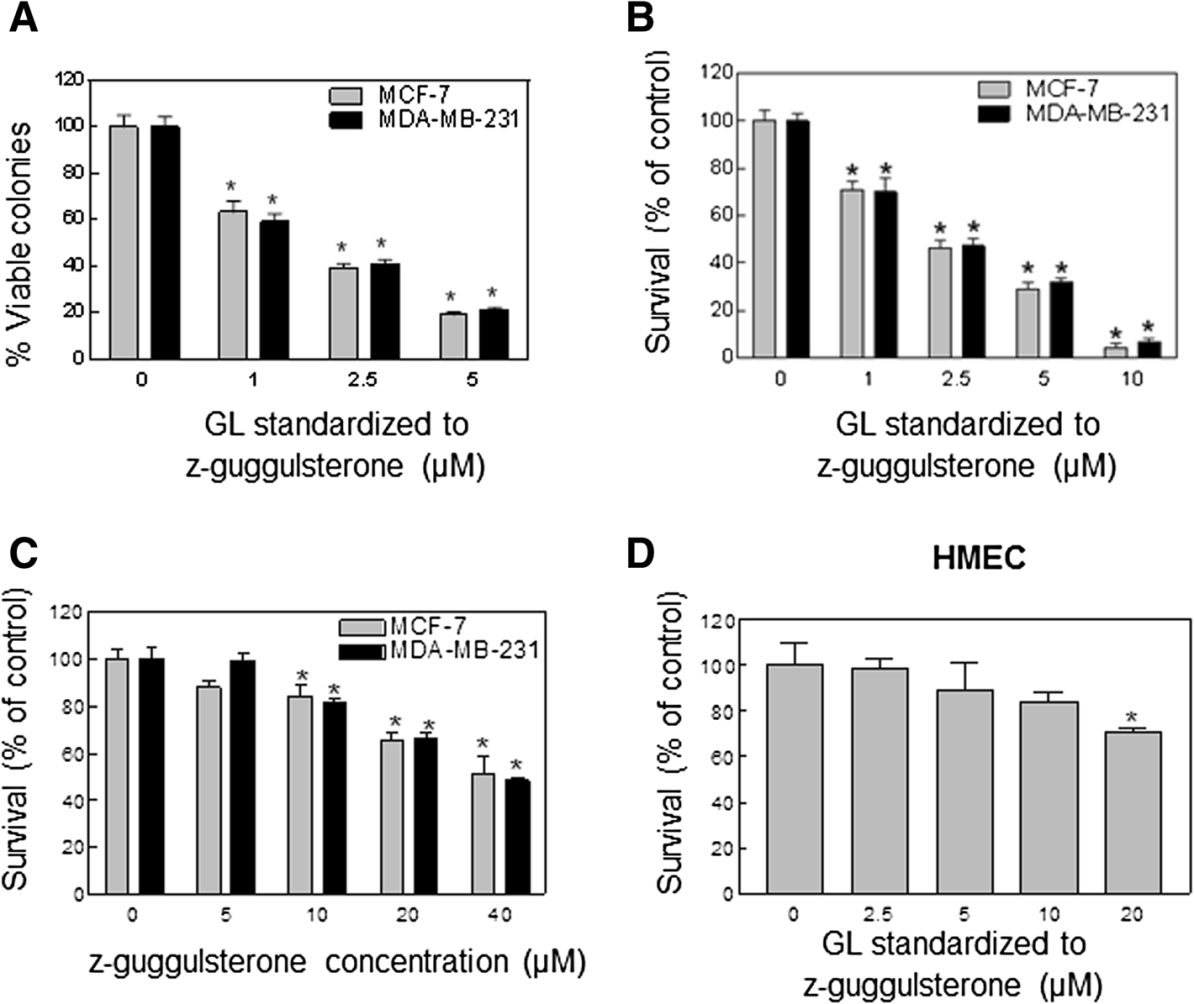
**A, Effect of GL (GL contains ~3.75% z-Gug and was standardized to z-Gug (μM)) on survival of MCF-7 and MDA-MB-231 (A) cells determined by the colonogenic survival assay.** Cells were treated with different concentrations of GL for 24 h. *Columns,* mean of three determinations; *bars,* SE. *Significantly different (*P*<0.05) compared with DMSO-treated control by one-way ANOVA followed by Dunnett’s test. Similar results were observed in two independent experiments. Representative data from a single experiment are shown. Effect of GL **(B** and **D)** and z-Gug **(C)** on survival of MCF-7, MDA-MB-231 and HMEC cells determined by the trypan blue dye exclusion assay. Cells were treated with different concentrations of GL or z-Gug for 24 h. *Columns,* mean of three determinations; *bars,* SE. *Significantly different (*P*<0.05) compared with DMSO-treated control by one-way ANOVA followed by Dunnett’s test. Similar results were observed in two independent experiments. Representative data from a single experiment are shown.

Initially, the colonogenic assay was used to determine the effect of GL on cell viability. By following the colony formation assaying procedure, the cells were cultured for 12 days after 24 h exposure to GL and the colony formation (>50 cells/colony) was determined. The viability of both MCF-7 and MDA-MB-231 cells was decreased significantly in a concentration-dependent manner with an IC_50_ of GL ~2 μM (standardized to z-Gug), which is within the pharmacologically achievable concentrations (~3 μM, 25, Figure 1A). To confirm the growth inhibitory effect of GL, we used the trypan blue dye exclusion assay. The results indicated that treatment with GL for 24 h resulted in a significant reduction in cell viability in both of cancer cells (Figure 1B). Since z-Gug is one of the major active components of GL, we compared the anti-cancer activity of GL with that of z-Gug. z-Gug inhibited the human breast cancer cell growth with an IC_50_ of GL ~30 μM (Figure 1C). However, growth inhibitory effect of GL to the cancer cells was ~10-fold stronger compared with that of z-Gug (Figure 1). The results indicate that the anti-cancer effect of GL on breast cancer cells is most likely attributable to z-Gug as well as other constituent(s). Interestingly, a normal human mammary epithelial cell line HMEC was significantly more resistant to growth inhibition by GL than were breast cancer cells (Figure 1D). For instance, 10 μM GL, which inhibited the viability of MCF-7 and MDA-MB-231 cells by about 90% (Figure 1A-B), had minimal effect on HMEC cell viability (Figure 1D). These data indicated that human breast cancer cells, but not HMEC, were sensitive to inhibition of cell viability by GL. Since the MCF-7 and MDA-MB-231 cells exhibited comparable sensitivity, we also concluded that estrogen receptor expression does not affect GL-mediated growth inhibition in human breast cancer cells.

### GL inhibits breast cancer cell growth by inducing apoptotic cell death

**Figure 2:**
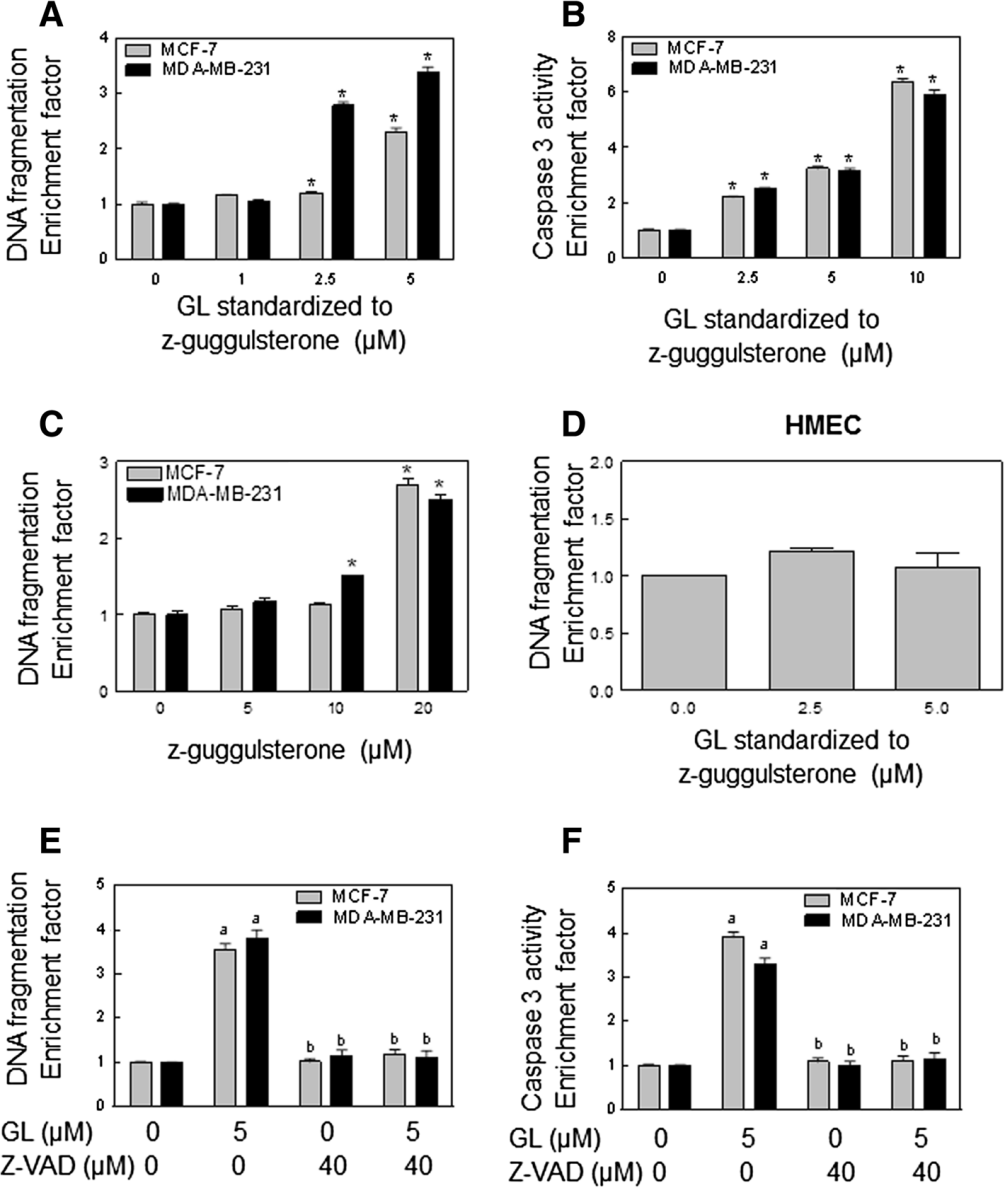
**GL induced apoptosis in MCF-7 and MDA-MB-231 cells, but not in HMEC, determined by (A and D) quantitation of cytoplasmic histone associated DNA fragmentation, and (B) flow cytomitry analysis of Caspase 3 activity.** Cells were treated with the indicated concentrations of GL or z-Gug **(C)** or DMSO (control) for 24 hours. Results are expressed as enrichment factor relative to cells treated with DMSO (control). Results are mean±SE (*n*=3). *Significantly different (*P*<0.05) between the indicated groups by one-way ANOVA followed by by Dunnett’s test. Similar results were observed in at least two independent experiments. Representative data from a single experiment are shown. Effect of pretreatment with general caspase inhibitor Z-VAD on GL-induced cytoplasmic histone-associated DNA fragmentation **(E)** and caspase 3 activity **(F)** in MCF-7 and MDA-MB-231 cells. Columns, mean (n=3); bars, SE. ^a^, p<0.05, significantly different compared with control; ^b,^ p<0.05, significantly different compared with GL alone treatment group (one-way ANOVA followed by Bonferroni’s test for multiple comparisons).

To gain further insights into the mechanism of GL-mediated inhibition of breast cancer cell growth, we determined its effect on cytoplasmic histone-associated DNA fragmentation, a widely used technique for detection of apoptosis. The GL treatment resulted in a dose-dependent increase in cytoplasmic histone-associated DNA fragmentation in both MCF-7 and MDA-MB-231 cells (Figure 2A). To confirm the results of GL-induced apoptotic cell death, we further investigated whether GL treatment increased caspase-3 activity in both cancer cells. A dose-dependent increase of caspase-3 activity was observed in GL-treated MCF-7 and MD-MB-231 cells (Figure 2B). Taken together, these observations clearly indicated that the antiproliferative effect of GL against breast cancer cells was associated with apoptosis induction (Figure 2A-B). Consistent with the cell viability data (Figure 1C), z-Gug at 20 μM can induce apoptosis (cytoplasmic histone-associated DNA fragmentation) in both cancer cells (Figure 2C). The same treatment with 2.5 and 5 μM GL to HMEC did not cause any apoptotic cell death (Figure 2D). The present study indicated that the effect of apoptosis inducing by GL is selective for breast cancer cells but not for normal human mammary epithelial cell HMEC (Figure 2D).

### GL-induced apoptosis is caspase-dependent

Next, we investigated whether the apoptotic cell death induced by GL is caspase dependence. To determine the role of caspases, the MCF-7 and MDA-MB-231 cells were pretreated with pan-caspase inhibitor Z-VAD 40 μM for 2 hours, and then were treated with 5 μM GL for 24 hours. As shown in Figure 2E-F, the GL-induced cytoplasmic histone-associated DNA-fragmentation and Caspase 3 activity in human breast cancer cells were fully blocked in the presence of Z-VAD. These results clearly suggest that the GL-induced apoptosis in human breast cancer cells was mediated by caspases.

### GL treatment caused the decrease of β-Catenin level in breast cancer cells

**Figure 3:**
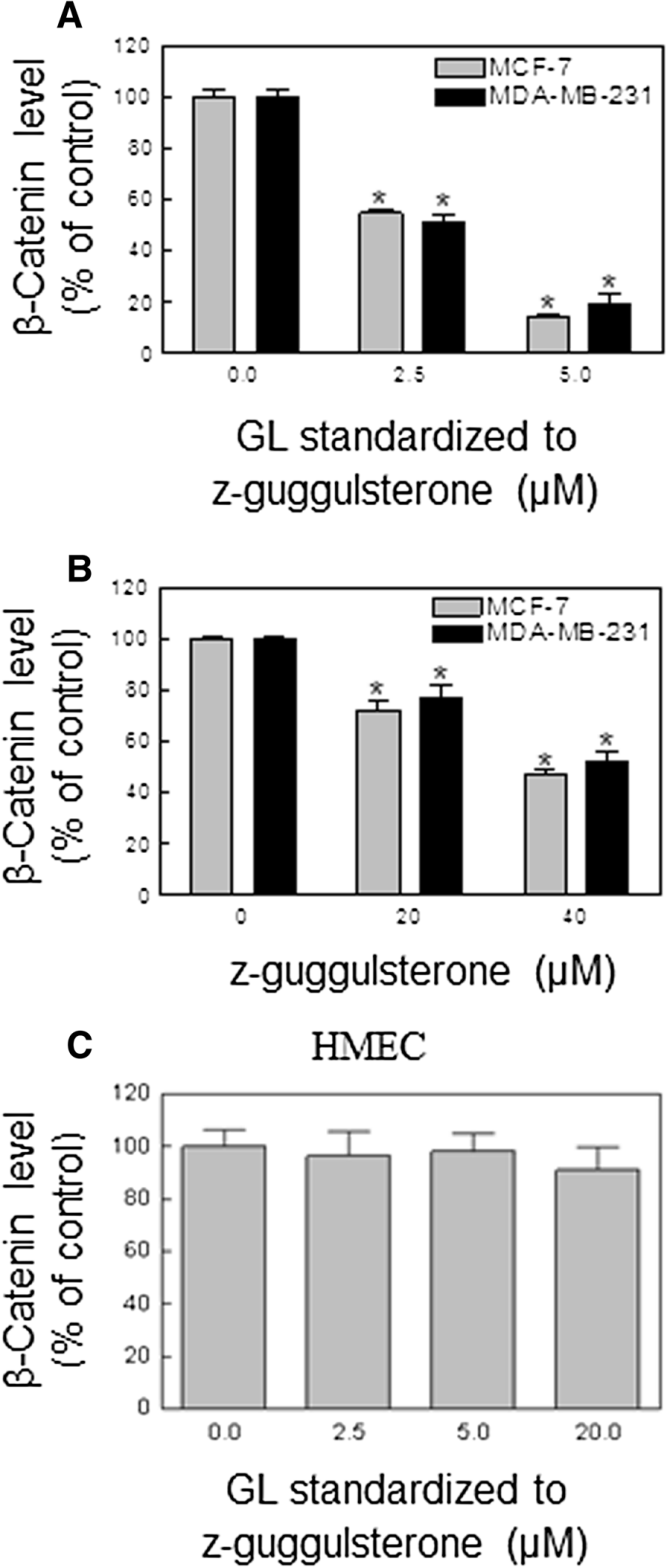
**GL (A and C) and z-Gug (B) inhibited β-Catenin level in human breast cancer MCF-7 and MDA-MB-231 cells as well as HMEC.** Cells were treated with DMSO (control) or 2.5 and 5 μmol/L GL standardized to z-Gug or 20 and 40 μmol/L z-Gug for 24h. β-Catenin level was determined by using the Surveyor^TM^ IC Human total β-Catenin Immunoassay kit (R&D Systems) following the manufacturer’s instructions. Experiments were repeated twice with triplicate measurements in each experiment. Results are mean±SE (*n*=3). *Significantly different (*P*<0.05) between the indicated groups by one-way ANOVA followed by Dennett’s test. The results were consistent and representative data from a single experiment are shown.

The Wnt/ β-Catenin signaling pathway plays an important role in carcinogenesis and tumor metastasis, which is involved in virtually many cancer types including breast cancer [29,30]. Thus, targeting the Wnt/ β-Catenin-TCF signaling is of great significance for chemoprevention and chemotherapy of cancer. The previous studies have shown that many natural products [31-35], such as, resveratrol [32], curcumin [33], cucurbitacin B [34], and lycopene [35] cause apoptosis through regulation of Wnt/ β-Catenin-TCF signaling. We tested whether GL-induced apoptosis was involved in β-Catenin signaling. As shown in Figure 3A, treatment with GL at 2.5 and 5 μM resulted in a dose-dependent decrease of the β-Catenin levels in human breast cancer MCF-7 and MDA-MB-231 cells compared with DMSO-treated (control) cells. For example, the β-Catenin levels in MCF-7 and MDA-MB-231 cells treated for 24 h with 5 μM GL were decreased by about 89% and 83%, respectively, as compared to the levels in the control group (Figure 3A). Significant inhibition of β-Catenin levels was also observed in the same time treatment of z-Gug at 20 and 40 μM in both cancer cells (Figure 3B). However, the GL-induced the decrease of the β-Catenin levels could not be observed in the GL-treated HMEC (Figure 3C). These observations clearly indicated that GL treatment resulted in the inhibition of β-Catenin signaling production in human breast cancer cells.

### Effect of GL treatment on the protein expression of β-Catenin and its targeting proteins in breast cancer cells

**Figure 4:**
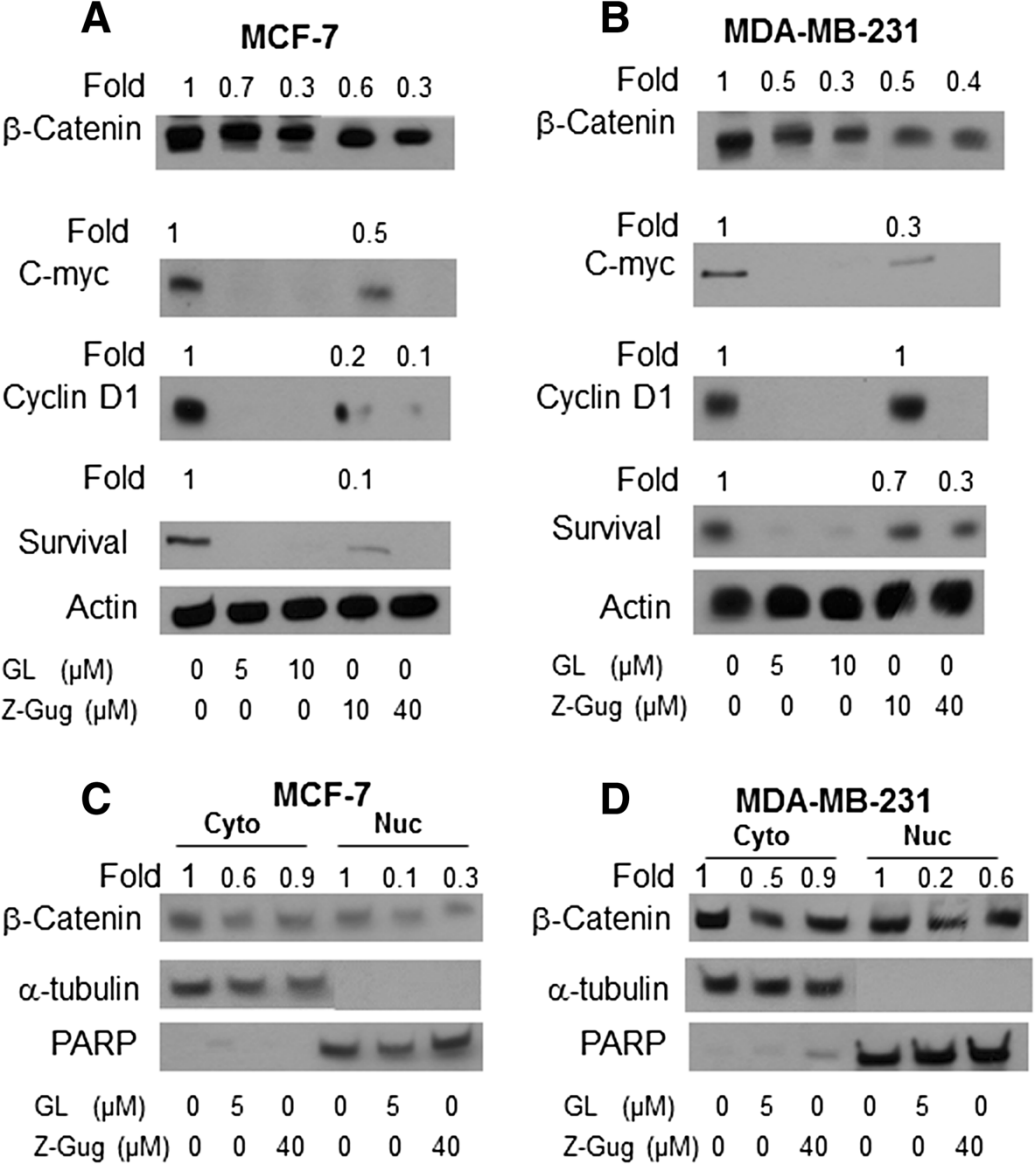
**Immunoblotting for β-Catenin, C-myc, Cyclin D1 and Survival proteins using lysates from MCF-7 (A) or MDA-MB-231 (B) cells treated with DMSO (control) or 5 and 10 μmol/L GL standardized to z-Gug or 10 and 40 μmol/L z-Gug for 24 h.** The blot was stripped and reprobed with anti-actin antibody to ensure equal protein loading. The numbers on top of the immunoreactive bands represent change in protein levels relative to corresponding DMSO-treated control. Immunoblotting for β-Catenin protein using isolated cytosolic and nuclear fractions from MCF-7 **(C)** or MDA-MB-231 **(D)** cells following 24-h treatment with DMSO or 5 μmol/L GL or 40 μmol/L z-Gug. The blot was reprobed with anti-α-Tubulin or anti-PARP antibody to ensure purity of the nuclear fraction. The numbers on top of the immunoreactive bands represent change in levels relative to DMSO-treated control. Immunoblotting for each protein was performed at least twice using independently prepared lysates.

To confirm the GL-inhibited effect of β-Catenin signaling, we determined whether GL treatment can regulate the β-Catenin protein levels. The effect of GL treatment on levels of β-Catenin protein in human breast cancer MCF-7 and MDA-MB-231 cells was determined by immunoblotting. Representative blots are shown in Figure 4A-B. The levels of β-Catenin protein were remarkably decreased in GL-treated MCF-7 and MDA-MB-231 cells. For example, MCF-7 cells treated with 5 and 10 μM GL resulted in a decrease of 30% and 70% for β-Catenin protein expression respectively, as compared with the controls (Figure 4A). The MDA-MB-231 cells had almost the same response to GL treatment (Figure 4B). To determine whether GL affects the protein expression of β-Catenin targeting genes, the protein levels of C-myc, Cyclin D1 and survivin were measured by immunobletting. GL treatment resulted in a significant down-regulation of these gene proteins (Figure 4A-B). In addition, z-Gug could inhibit the protein expression of β-Catenin as well as the protein levels of C-myc, Cyclin D1 and survivin in both cancer cells (Figure 4A-B). These results indicated that GL treatment inhibited the β-Catenin and its targeting gene proteins in human breast cancer cells.

### GL inhibited the nuclear translocation of β-Catenin

Since GL treatment down-regulated the β-Catenin signaling pathway in the human breast cancer cells (Figures 3– 4), we hypothesized that GL might also inhibit translocation of β-Catenin to nuclei. To determine the β-Catenin protein levels in both the cytoplasmic and nuclear fractions, we isolated both fractions from the control cells and the GL- or z-Gug-treated cells. The expression of β-Catenin protein in the cytoplasmic and nuclear fractions was measured by immunoblotting (Figure 4C-D). GL as well as z-Gug treatments significantly decreased the β-Catenin protein level of the nuclear fraction in both MCF-7 and MDA-MB-231 cells (Figure 4C-D). The treatment of 5 μM GL greatly reduced the level of nuclear β-Catenin in both cancer cells. For example, GL down-regulated the nuclear β-Catenin protein by 80%-90% with respect to the protein level in the the control cells (Figure 4C-D). These data suggested that GL inhibited the nuclear translocation of β-Catenin protein in human breast cancer cells.

### GL-induced apoptosis was enhanced by RNA interference of β-Catenin in breast cancer cells

**Figure 5:**
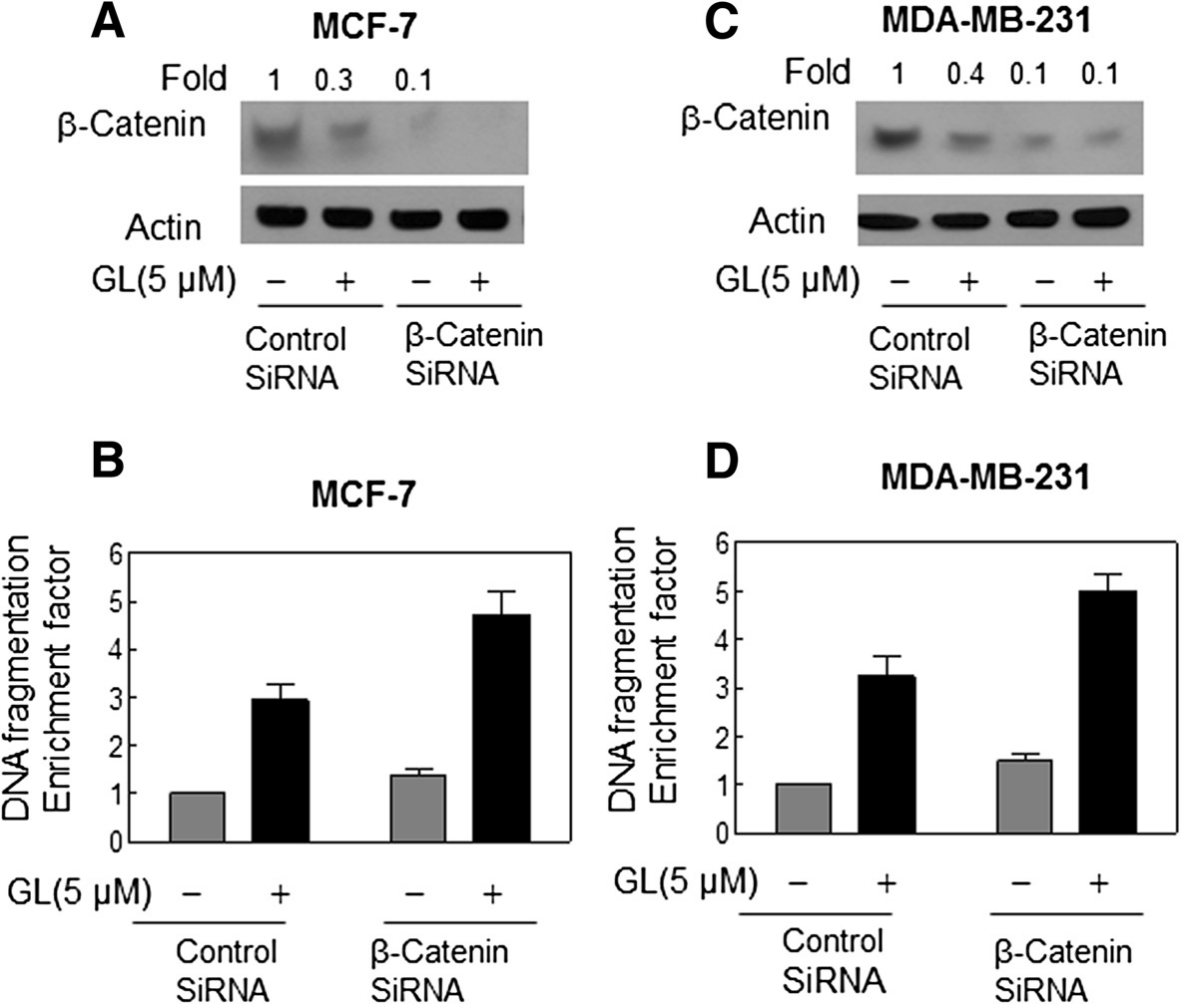
**Immunoblotting for β-Catenin using lysates from MCF-7 (A) or MDA-MB-231 (C) cells transiently transfected with a control nonspecific siRNA or β-Catenin -targeted siRNA and treated for 24 h with DMSO or 5 μmol/L GL.** The blots were stripped and reprobed with anti-actin antibody to ensure equal protein loading. The numbers on top of the immunoreactive bands represent changes in protein levels relative to DMSO-treated nonspecific control siRNA–transfected cells. Cytoplasmic histone-associated apoptotic DNA fragmentation in MCF-7 **(B)** or MDA-MB-231 **(D)** cells transiently transfected with a control nonspecific siRNA or β-Catenin -targeted siRNA and treated for 24 h with DMSO or 5 μmol/L GL. The results are expressed as enrichment factor relative to DMSO-treated control cells transiently transfected with the control nonspecific siRNA. Each experiment was done twice, and representative data from a single experiment are shown. Columns, mean (*n*=3); bars, SE. *, *P*<0.05, significantly different between the indicated groups by paired *t* test.

Next, we used siRNA technology to directly test the contribution of β-Catenin in the regulation of GL-induced apoptosis. As shown in Figure 5A-B, the level of β-Catenin protein was decreased by∼90% by transient transfection of MCF-7 and MDA-MB-231 cells with β-Catenin-targeted siRNA compared with cells transfected with a control nonspecific siRNA. Similar to GL’s effect on untransfected cells (Figure 4A-B), the GL treatment (5 μmol/L, 24 h) caused a decrease in levels of β-Catenin in both MCF-7 and MDA-MB-231 cells transfected with the nonspecific control-siRNA (Figure 5A and C). The GL-mediated reductions of β-Catenin were markedly enhanced in both MCF-7 and MDA-MB-231 cells transfected with the β-Catenin -targeted siRNA (Figure 5A and C). Twenty-four–hour exposure of nonspecific control-siRNA–transfected MCF-7 and MDA-MB-231 cells to 5 μM GL resulted in around 3-fold increase in cytoplasmic histone-associated DNA fragmentation compared with DMSO-treated control (Figure 5B and D). A remarkable increase apoptotic cell death was observed in both β-Catenin-depleted cells treated with GL compared with cells transfected with control-siRNA and treated with GL (Figure 5B and D). Collectively, these results indicated that β-Catenin play an important role in the GL-induced apoptosis in human breast cancer cells.

### GL inhibited the β-Catenin-medicated TCF protein expression and the knockdown of TCF-4 protein increased GL-induced apoptotic cell death in human breast cancer cells

**Figure 6:**
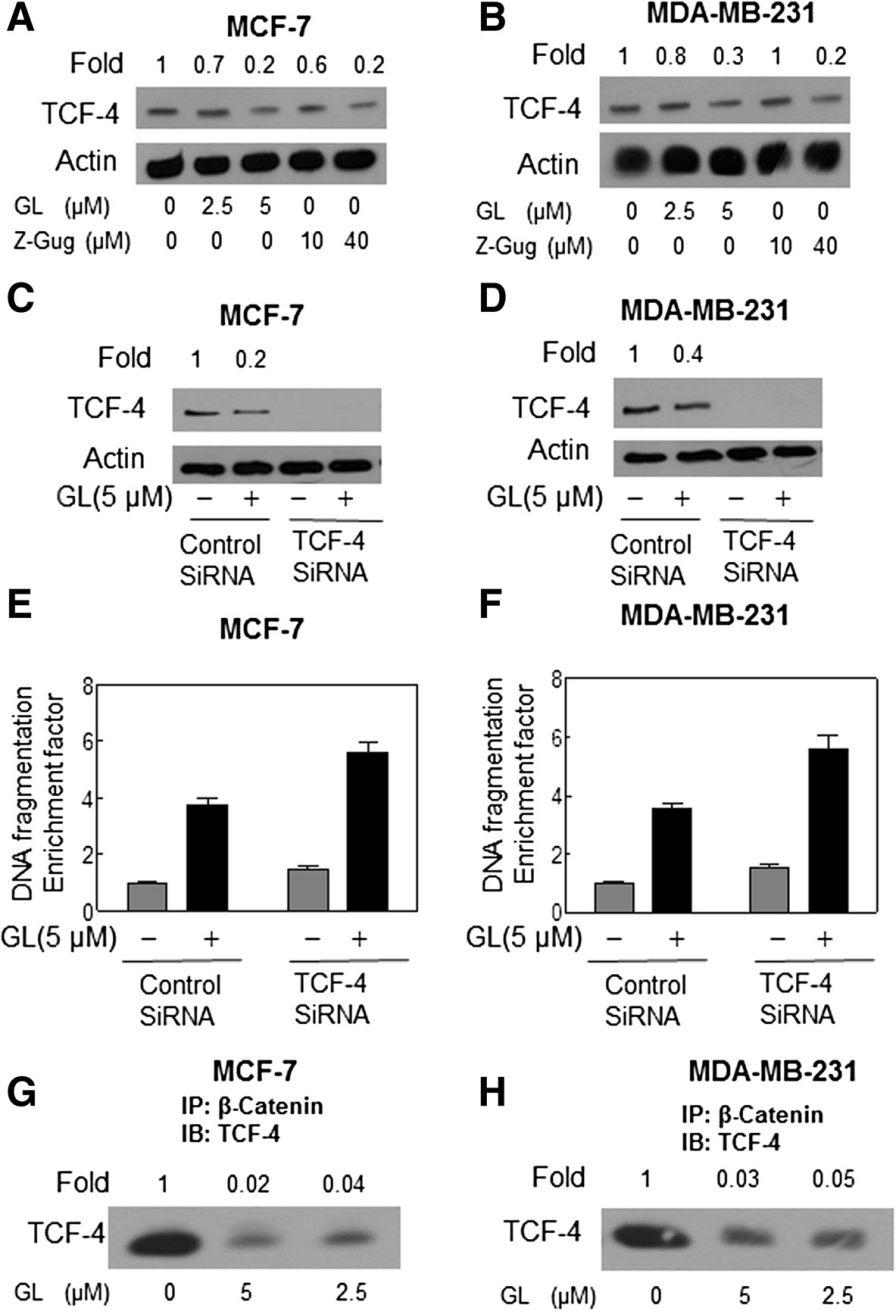
**Immunoblotting for TCF-4 using lysates from MCF-7 (A) or MDA-MB-231 (B) cells treated with DMSO (control) or 5 μmol/L GL for 24 h.** Immunoblotting for TCF-4 using lysates from MCF-7 **(C)** or MDA-MB-231 **(D)** cells transiently transfected with a control nonspecific siRNA or TCF-4 -targeted siRNA and treated for 24 h with DMSO or 5 μmol/L GL. The blots were stripped and reprobed with anti-actin antibody to ensure equal protein loading. The numbers on top of the immunoreactive bands represent changes in protein levels relative to DMSO-treated control cells **(A-B)** or relative to DMSO-treated nonspecific control siRNA–transfected cells **(C-D)**. Cytoplasmic histone-associated apoptotic DNA fragmentation in MCF-7 **(E)** or MDA-MB-231 **(F)** cells transiently transfected with a control nonspecific siRNA or TCF-4-targeted siRNA and treated for 24 h with DMSO or 5 μmol/L GL. The results are expressed as enrichment factor relative to DMSO-treated control cells transiently transfected with the control nonspecific siRNA. Each experiment was done twice, and representative data from a single experiment are shown. Columns, mean (*n*=3); bars, SE. *, *P*<0.05, significantly different between the indicated groups by paired *t* test. GL treatment inhibited β-Catenin binding to TCF in human breast cancer cells. Immunoblotting for TCF-4 using β-Catenin immunoprecipitates from MCF-7 **(H)** and MDA-MB-231 **(G)** cells treated for 24 h with DMSO (control) or GL 2.5 and 5 μM. The numbers on top of the immunoreactive bands represent change in levels relative to DMSO-treated control for each cell line. Each experiment was performed at least twice using independently prepared lysates.

Since β-Catenin is involved in the GL-induced apoptosis, we questioned whether GL-induced apoptotic cell death is regulated by β-Catenin/TCF signaling. To elucidate the mechanism of GL-induced apoptosis in human breast cancer cells, we investigated its effect on TCF protein expression. The MCF-7 (Figure 6A) and MDA-MB-231 (Figure 6B) cells with 2.5 and 5 μM GL exhibited a reduction of TCF protein level. In addition, treatment of 40 μM z-Gug were found to down-regulate the expression of TCF protein in both MCF-7 and MDA-MB-231 cells (Figure 6A-B). These results indicated that the TCF signaling may be involved in GL-induced apoptosis in human breast cancer cells. Therefore, the role of the TCF signaling in the apoptosis induction by GL was determined by using the siRNA technology. As can be seen from Figure 6C-D, the protein levels of TCF were nearly knocked down in both MCF-7 and MDA-MB-231 cells by transient transfection with TCF-4-targeted siRNA when compared with cells transfected with a nonspecific control-siRNA. Furthermore, exposure to GL 5 μM for 24 h resulted in statistically significant increase in cytoplasmic histone-associated DNA fragmentation (around 4 fold of control) in the control-siRNA transfected MCF-7 and MDA-MB-231 cells, but resulted significantly greater increase (around 6 fold of control) in the cells transiently transfected with the TCF-4-siRNA (Figure 6E-F). The data indicated that the GL-induced apoptosis is mediated by β-Catenin/TCF signaling pathways.

### GL inhibited the binding of β-Catenin to TCF-4 in human breast cancer cells

Previous studies have shown that nuclear β-Catenin complexes with TCF-4 and disruption of this interaction are necessary for the inhibition of β-Catenin/TCF-4 signaling pathway [31,32]. Therefore, we tested the possibility of whether GL treatment affected the interaction between β-Catenin and TCF-4. As shown in Figure 6G-H, the β-Catenin-TCF-4 complex was detectable in DMSO-treated (control) MCF-7 and MDA-MB-231 cells as evidenced by immunoprecipitation using the anti- β-Catenin antibody followed by immunoblotting with the anti-TCF-4 antibody. The level of β-Catenin-TCF-4 complex was decreased by ∼95% in MCF-7 and MDA-MB-231 cells treated with 2.5 and 5 μM GL for 24 h when compared with that of the DMSO-treated cells (Figure 6G-H). These results showed that GL treatment disrupted the interaction between β-Catenin and TCF-4 in human breast cancer cells.

## Discussion

GL has recently been the focus of attention for its chemopreventive and chemotherapeutic potential for cancer [10-24]. Although the mechanisms of the anti-cancer action of GL are not completely understood, these studies, including ours, have indeed indicated that GL and its active components (Gugs) inhibit cancer cell viability by causing apoptosis [10-24]. The mechanisms underlying GL-induced apoptosis are involved in the change of Bcl-2 gene family proteins [10,12-17], the inhibition of NF-kB signaling [17-19,22], the regulation of MAPK pathways [13,15], the suppression of farnesoid X receptor [5,18] and the bile acid receptor [5,18], and the inhibition of EGFR-STAT3 signaling [20,23]. The results implicated the involvement of the STAT3 and the VEGF/VEGFR signaling pathways in the regulation of GL-mediated anti-angiogenesis activity [14,23]. In the present study, we, for the first time, showed that GL treatment significantly inhibited the human breast cancer cell growth and caused apoptotic cell death. Our results revealed a novel anti-cancer mechanism of GL, in which the β-Catenin signaling pathway is involved in the GL-induced apoptosis in human breast cancer.

Our data indicate that GL has a stronger anti-cancer potential in human breast cancer cells than one of its active constituents (z-Gug) as evidenced by their differences in the inhibition of cell growth and the induction of apoptotic cell death. The inhibition of cell survival by GL was Statistically significant as evidenced by IC_50_~2 μmol/L concentrations standardized to z-Gug. The growth inhibition by GL was ~10-fold stronger compared with that of z-Gug (Figure 1). although pharmacokinetic parameters for GL have not been determined in humans, the maximal plasma concentration of z-Gug (*C*_max_) in rats was shown to be 3.3- and 18.3 μmol/L following oral gavage with 50 mg z-Gug/Kg body weight and intravenous injection with 18 mg z-Gug/Kg body weight, respectively [25]. Based on these pharmacokinetic observations, it is possible that the concentrations of GL needed to inhibit cancer cell growth may be achievable in humans. Our previous results have shown that a normal prostate epithelial cell line PrEC was significantly more resistant to growth inhibition by GL or z-Gug compared with prostate cancer cells. Therefore, we investigated whether GL treatment has a selective activity to human breast cancer calls and human normal mammary epithelial cells by conducting experiments on the effects of GL on the proliferation and apoptosis inducing of HMEC, a normal human mammary epithelial cell line. The data clearly showed that GL-treated HMEC cells displayed a relatively significantly less cell growth inhibition than the GL-treated human breast cancer cell (Figure 1). Based on these results, we conclude that (a) uncharacterized constituent(s) of GL may interact additively or synergistically to inhibit the viability of human breast cancer cells, (b) a normal human mammary epithelial cell line is significantly more resistant to the growth inhibition by GL, and (c) the growth inhibition of human breast cancer cells by GL is not influenced by the estrogen receptor expression.

The present results clearly indicated that anticancer activity of GL against human breast cancer cells is associated with apoptosis induction. The conclusion is based on the following: (a) GL treatment caused a significant increase of cytoplasmic histone-associated DNA fragmentation in human breast cancer cells (Figure 2A), (b) an increase of caspase 3 activity was observed in GL-treated breast cancer cells (Figure 2B), and (c) the same GL treatment could not induce apoptosis in the normal human mammary epithelial cell HMEC (Figure 2D).

The Wnt/β-Catenin signaling is involved in the development and disease including cancer [29,30,36]. Deregulation of the Wnt/β-Catenin signaling has been implicated in the pathogenesis of many kinds of human cancers including breast cancer [29-31,34-36]. Since β-Catenin plays a key role in the Wnt signaling pathway [29,36], the disruption of Wnt/β-Catenin signaling represents a great opportunity to develop novel drugs for cancer chemoprevention and therapy [31-37]. Some natural products have shown the potential for the inhibition of cancer growth through the inactivation of the Wnt/β-Catenin signaling [31-37]. For example, resveratrol was capable of disrupting the binding between β-Catenin and TCF-4, but not affect the accumulation and nuclear targeting of β-Catenin signaling [32]. The molecular mechanism of curcumin for the anticancer action is involved in the inhibition of nuclear β-Catenin transcription activity and the enhancement of the levels of membrane β-Catenin via the activation of PKD1 [33]. Cucurbitacin B induced apoptosis and caused the growth inhibition of breast cancer cells through the reduction of the Wnt-associated proteins and the inhibition of GSK-3 β-mediated β-Catenin to the nucleus [34]. Lycopene synergistically enhanced quinacrine anticancer action and inhibited the β-Catenin signaling is dependent to APC [35]. Our present results indeed suggest that the β-Catenin signaling pathway is involved in the GL-induced breast cancer cell growth inhibition and apoptosis inducing. First of all, the β-Catenin levels were significantly reduced in GL-treated human breast cancer MCF-7 and MDA-MB-231 cells compared to DMSO-treated control cells (Figure 3). The data were confirmed by the immunblotting results that showed the GL treatment resulted in a dose-dependent down-regulation of the β-Catenin protein expression in both cancer cells (Figure 4A-B). Furthermore, a significant inhibition of the nuclear translocation of β-Catenin was observed in both cancer cells that were treated with 5 μM GL and 40 μM z-Gug (Figure 5C-D). To determine the real role of β-Catenin in the GL-induced apoptotic cell death in our models, we knocked-down β-Catenin in the cells by β-Catenin-siRNA and then measured the β-Catenin protein expression and apoptosis induction. The present data showed that the GL-induced down-regulation of β-Catenin and apoptosis were significantly enhanced by the siRNA knock-down in the both cancer cells (Figure 5). Taken together, these results indicated that β-Catenin is the real target for the GL-induced apoptosis in human breast cancer cells.

The Wnt/β-Catenin signaling plays an important role in breast cancer initiation, progression and metastasis [30,38-41]. The activation status of Wnt/ β-Catenin signaling has been reported in human breast cancer [29,30,38-41]. β-Catenin has a central role in Wnt signaling pathway via effects on the TCF-mediated transcription [29-31]. The abnormal β-Catenin levels could enhance the β-Catenin binding to TCF [29-31,38-41]. We hypothesized that the apoptosis response to GL may be mediated by TCF-4. Herein, we have provided the evidence that the GL treatment causes significant down-regulation of the TCF-4 protein expression in human breast cancer MCF-7 and MDA-MB-231 cells (Figure 6A-B). Moreover, the GL-induced apoptotic cell death as well as the reduction of TCF-4 levels is significantly enhanced by the knockdown of TCF-4 (Figure 6C-F). Therefore, we conclude that the GL-induced apoptotic cell death in human breast cancer cells is regulated by TCF-4. Importantly, our present results showed that the GL treatment reduced a ∼ 95% of the complex of β-Catenin/TCF in MCF-7 and MDA-MB-231 cells, compared with the DMSO-treated control cells (Figure 6H-G). In conclusion, GL treatment does not only inhibit the nuclear translocation of β-Catenin and the TCF-4 protein expression but also disrupts the β-Catenin/TCF-4 complex in the human breast cancer cells (Figures 5 and 6). For example, treatment of 5 μM in MCF-7 and MDA-MB-231 cells down-regulated the β-Catenin protein expression by 50-60% and the TCF-4 protein levels by 60-70%, respectively (Figures 4– 6). The same treatment, however, can exert a ∼ 95% inhibition of the β-Catenin binding to TCF-4 in both cancer cells (Figure 6).

A variety of Wnt/ β-Catenin targeting genes have been identified, including those that regulate cell proliferation and apoptosis, thus mediating cancer initiation and progression [29,36]. TCF-4 binds to β-Catenin to transactivate Wnt target genes that include oncogenic genes c-Myc, cyclin D1 and survivin [29-31,38-41]. Interestingly, we found that GL and z-Gug significantly inhibit the protein expression of Wnt/β-Catenin downstream effectors c-Myc, cyclin D1 and survivin in both breast cancer cells (Figure 4A-B).

## Conclusion

We, for the first time, reported that GL is a potent inhibitor of breast cancer cell growth. The present study reveals a novel mechanism of GL-anticancer activity. The GL-induced apoptotic cell death in human breast cancer is regulated by the β-Catenin signaling pathway.

## Abbreviations

GL: Gugulipid; z-Gug: z-guggulsterone; Gugs: Guggulsterones; DMSO: Dimethyl sulfoxide; PBS: Phosphate buffered saline; HMEC: Normal human mammary breast epithelial cell line.

## Competing interests

All authors declare that they have no competing interests.

Sabinsa Corporation supplied Gugulipid and provided the analytical results on the Guggulsterones for the study. Sabinsa Corporation did not influence in anyway the interpretation of the results nor did provide any grant or funds to carry out the study. Hence Sabinsa Corporation declares to have no competing interests in the publication of this study.

## Authors’ contributions

GQJ and DX provided oversight for the project, conducted the experiments and wrote the manuscript. XX and YZ performed the experiments, participated in data analysis and the manuscript preparation. KN and MM provided gugulipid and carried out quality control. All authors read and approved the final manuscript.

## Pre-publication history

The pre-publication history for this paper can be accessed here:

http://www.biomedcentral.com/1472-6882/13/203/prepub
